# The embodiment of emotion-label words and emotion-laden words: Evidence from late Chinese–English bilinguals

**DOI:** 10.3389/fpsyg.2023.1143064

**Published:** 2023-03-22

**Authors:** Dong Tang, Yang Fu, Huili Wang, Bo Liu, Anqi Zang, Tommi Kärkkäinen

**Affiliations:** ^1^School of Foreign Languages, Dalian University of Technology, Dalian, China; ^2^Faculty of Information Technology, University of Jyväskylä, Jyväskylä, Finland; ^3^Instituto Universitario de Neurociencia, Universidad de La Laguna, Santa Cruz de Tenerife, Spain; ^4^School of Foreign Languages, Hangzhou City University, Hangzhou, China; ^5^School of Foreign Languages, Dalian Maritime University, Dalian, China

**Keywords:** emotion-label words, emotion-laden words, emotion word type, valence, embodied cognition

## Abstract

Although increasing studies have confirmed the distinction between emotion-label words (words directly label emotional states) and emotion-laden words (words evoke emotions through connotations), the existing evidence is inconclusive, and their embodiment is unknown. In the current study, the emotional categorization task was adopted to investigate whether these two types of emotion words are embodied by directly comparing how they are processed in individuals’ native language (L1) and the second language (L2) among late Chinese-English bilinguals. The results revealed that apart from L2 negative emotion-laden words, both types of emotion words in L1 and L2 produced significant emotion effects, with faster response times and/or higher accuracy rates. In addition, processing facilitation for emotion-label words over emotion-laden words was observed irrespective of language operation; a significant three-way interaction between the language, valence and emotion word type was noted. Taken together, this study suggested that the embodiment of emotion words is modulated by the emotion word type, and L2 negative emotion-laden words tend to be affectively disembodied. The disassociation between emotion-label and emotion-laden words is confirmed in both L1 and L2 and therefore, future emotion word research should take the emotion word type into account.

## 1. Introduction

The role of emotion in grounding conceptual-semantic representations during language processing should not be underestimated (Kousta et al., 2011). According to the traditional amodal theory, concepts are represented with abstract and arbitrary mental symbols, without the involvement of specific modalities (Fodor, 1975; Charniak, 1978). However, this disembodied account has been challenged by embodied cognition, a recent dominant view that posits that the comprehension of language is grounded in bodily perception, action, as well as emotion (for reviews: Horchak et al., 2014; Kühne and Gianelli, 2019). Accumulating evidence supporting this embodied account has reported that the processing of sensory or action-related linguistic items involves reactivation of the same neural mechanism as one executes a specific action (for a review: Fischer and Zwaan, 2008). In this line, given that emotion words carry a large emotional load, many studies have been conducted to examine the relationship between language and emotion. There is evidence suggesting that emotion words produce emotion activation automatically and are therefore embodied. For example, it has been reported that emotion words are processed with faster reaction times (Kousta et al., 2009) and increased neural correlates (Citron, 2012) compared to neutral words, and this processing advantage of emotion words is known as the “emotion effect” (Wang et al., 2019). Additionally, neuroscientific studies have revealed that the original sensory-motor and emotion-related regions get activated when participants are exposed to concepts with emotion-evoking content (e.g., Moseley et al., 2012). These findings suggested that language understanding is grounded in emotion simulation.

However, the emotion word processing in bilingualism poses a challenge to embodied cognition. It has been proposed that emotion words in a native language (L1) are more embodied compared to those in the second language (L2) (e.g., Baumeister et al., 2017). This is because L1 emotion words are more closely linked to specific contexts or situations where sensory-motor experiences and linguistic concepts are established. As a result, when encountering L1 emotion words, emotional experiences associated with those words are reactivated, thereby contributing to language understanding. In contrast, there is a greater emotional distance in L2 (Pavlenko, 2012), and therefore it remains open with respect to whether emotion words in L2 are embodied. Some studies revealed that emotion activation in response to L2 emotion words is similar to that in L1 (e.g., Ponari et al., 2015; Sheikh and Titone, 2016), whereas others suggested that emotion words in L2 may not activate or only weakly activate emotions compared to L1 emotion words (Conrad et al., 2011; Degner et al., 2012), especially for those acquired in adult age (Kühne and Gianelli, 2019).

Recently, an additional issue has emerged in the field of emotion word research regarding the precise definition of emotion words. Usually, two emotional dimensions, including valence (pleasant or unpleasant; positive or negative category of emotional stimuli) and arousal (calm or excited; low or high degree of emotion activation), were primarily explored in prior research (Hinojosa et al., 2020). However, critics have pointed out that in much of the prior research on emotion word processing, the emotion word type was not taken into account (Altarriba and Basnight-Brown, 2011; Zhang et al., 2017). Accordingly, emotion words can be categorized into two subtypes: emotion-label words (e.g., “happy,” “sad”) which straightforwardly elucidate or describe one’s affective states, and emotion-laden words (e.g., “successful,” “failed”) which elicit individual’s emotions through the word’s connotations (Pavlenko, 2008; Zhang et al., 2017). The “emotion word type effect,” which refers to the disassociation between these two kinds of emotion words (Wu and Zhang, 2019; Wu et al., 2021b), has been confirmed in an increasing number of studies. Specifically, in terms of monolingual research, the emotion word type effect was observed in behavioral studies with various cognitive tasks (Knickerbocker and Altarriba, 2013; Kazanas and Altarriba, 2015, 2016b; El-Dakhs and Altarriba, 2019). For example, Kazanas and Altarriba (2015) found facilitated processing of emotion-label words in both implicit (masked) and explicit (unmasked) lexical decision task (LDT), with faster response times (RTs) and greater priming effects relative to emotion-laden words. Research using event-related potentials (ERPs) has further demonstrated different neural mechanisms underlying the processing of L1 emotion-label and emotion-laden words (Zhang et al., 2017, 2019b; Wang et al., 2019; Wu et al., 2020, 2021a,b; Li et al., 2022; Liu et al., 2022a,b; Yeh et al., 2022). Zhang et al. (2017) for instance, compared the time course of emotion activation of the two kinds of emotion words in an LDT. They found that emotion-label words elicited enhanced N170 on the right hemisphere in comparison to emotion-laden words, and negative emotion-label words elicited larger Late Positivity Complex (LPC) on the right hemisphere relative to that on the left hemisphere. In addition, such discrepancies between emotion-label and emotion-laden words have also been observed in L2 processing in behavioral (Altarriba and Basnight-Brown, 2011; Kazanas and Altarriba, 2016a; El-Dakhs and Altarriba, 2019; Bromberek-Dyzman et al., 2021) and ERPs studies (Wu et al., 2019; Wu and Zhang, 2019; Zhang et al., 2019a, 2020). For example, in an ERPs study (Zhang et al., 2020), the emotion word type effect was found as the two kinds of emotion words in L2 were identified divergently across early and late processing stages.

The behavioral and neural studies outlined above present converging evidence confirming the emotion word type effect. Motivated by these findings, the present study aims to investigate and compare the potential modulation of the emotion word type on the embodiment of emotion words in L1 and L2 processing, given distinct associations of these two types of emotion words with emotional states. However, certain concerns must be addressed regarding the existing research on emotion word type. One such concern is that there has yet to be a consensus on which type of emotion word has the processing advantage in L1 and L2. While some studies found facilitated processing for emotion-label words relative to emotion-laden words in either L1 (e.g., Kazanas and Altarriba, 2015; Zhang et al., 2017) or L2 (e.g., Wu et al., 2019), or both (e.g., El-Dakhs and Altarriba, 2019), Kazanas and Altarriba (2016a) in their bilingual study found such processing superiority of emotion-label words was only restricted to the dominant language of participants. In contrast, others reported processing facilitation for emotion-laden words. For example, behavioral data from the ERPs study of Zhang et al. (2020) showed the processing advantage for L2 emotion-laden words over emotion-label words among Chinese-English bilinguals. In a similar vein, Bromberek-Dyzman et al. (2021) found a processing advantage for emotion-laden words in both L1 and L2 with a valence decision task, associating with faster RTs and higher accuracy rates (ACCs).

In addition, there are methodological concerns regarding the stimulus characteristics in prior examinations on the emotion word type. One methodological concern is the failure to control the concreteness of experimental stimuli in some studies (e.g., Kazanas and Altarriba, 2016a; Zhang et al., 2017). For instance, although Zhang et al. (2017) demonstrated differences in ERPs in processing these two types of emotion words, they did not manage to control the concreteness of experimental stimuli. Notably, when words’ concreteness was strictly controlled, the differences in N170 and LPC components were not replicated in the study by Wang et al. (2019). Another methodological concern is that the lexical categories were intermixed (e.g., Kazanas and Altarriba, 2015; Zhang et al., 2017). For example, Kazanas and Altarriba (2015) used emotion-laden words that were all nouns (e.g., “candy,” “coffin”), while the emotion-label words consisted of adjectives (e.g., “happy,” “afraid”) and nouns (e.g., “delight,” “anger”). It is therefore still being determined whether the reported divergencies in processing emotion-label and emotion-laden words should be attributed to such methodological factors or the different types of emotion nature. More importantly, it is worth noting that other studies have shown no discrepancy between these two types of emotion words (Vinson et al., 2014; Martin and Altarriba, 2017). For instance, Martin and Altarriba (2017) found that emotion-label words were similarly processed with emotion-laden words as they produced similar response latency in an LDT employing hemifield presentation.

Therefore, there is no clear-cut answer regarding the emotion word type effect. In order to know the embodiment of emotion-label and emotion-laden words in L1 and L2, it is necessary to first determine whether a distinction between them exists, as well as which type of emotion word has a processing advantage. Another problem concerns the modulation of valence in emotion word processing. Although prior research has frequently reported the valence effect in emotion word processing, there is no consensus on whether it is positive or negative information that enhances word processing (Crossfield and Damian, 2021; for a review: Kauschke et al., 2019). For example, some studies found a positivity bias which shows that positive emotion words are responded to with faster response times (Goh et al., 2016), while others found the opposite pattern, a negative bias (e.g., Dijksterhuis and Aarts, 2003; Nasrallah et al., 2009). Given that the valence effect, for example, the positivity bias, has also been observed in processing these two types of emotion words (e.g., Kazanas and Altarriba, 2015, 2016a; El-Dakhs and Altarriba, 2019), the present study employed the emotional categorization task (ECT) in which the valence dimension is task-relevant. With this task, deep processing of emotion words is expected to be induced as participants internally simulate the emotional properties or contents of stimuli.

Furthermore, the existing behavioral research on the emotion word type in bilingualism was mainly conducted among Spanish-English bilinguals (e.g., see a series of studies conducted by Kazanas and Altarriba). However, emotions may be conceptualized divergently in different languages (Bromberek-Dyzman et al., 2021; Zhou et al., 2022). For instance, Zhou et al. (2022) pointed out that Chinese emotion words are embodied more interoceptive, while English emotion words are embodied more autonomic. This divergence may lead to Chinese speakers being more reflective and English speakers more proactive in emotional linguistic expressions. Murata et al. (2013) also found that Westerners are inclined to directly express what they feel as they value high-arousal emotions (emotional expression), whereas Asian people are culturally and historically trained to value low-arousal emotions (emotional control). Given these findings, the present study included the logographic L1 (Chinese) and alphabetic L2 (English) as the represented languages to investigate whether there are differences in processing L1 and L2 emotion words that directly name or indirectly evoke affective states among Chinese-English bilinguals.

Based on prior studies, we hypothesized (1) embodiment for both types of emotion words in L1 and L2, except for L2 emotion words with negative valence (Sheikh and Titone, 2016); (2) a processing advantage (faster RTs and higher ACCs) for words in L1 rather than in L2 (Chen et al., 2015), for words with positive valence rather than with negative valence, for emotion-label words rather than emotion-laden words in both L1 and L2, possibly with a more robust emotion word type effect in participants’ dominant language (Chinese) than their non-dominant language (English) (Kazanas and Altarriba, 2016a); (3) the modulation of valence and language on the two categories of emotion word processing with faster RTs and/or higher ACCs for positive emotion-label words compared to negative emotion-laden words in L1 and L2.

## 2. Methodology

### 2.1. Participants

Fifty-two postgraduate and doctoral students were recruited in this experiment (17 males, mean age: 27.69, SD = 3.55), with Chinese as their L1 and English as their L2. According to their self-reports, all participants were born and live in China. They began learning English as their L2 at the mean age of 9.87 years old and had an average of 17.83 years of English acquisition, suggesting that they are late bilinguals (Pavlenko, 2012). In addition, all participants were right-handed and had normal or corrected-to-normal vision without neurobiological or psychiatric disorders. Prior to the experiment, all participants were required to complete an English proficiency test (especially the lexical knowledge) named LexTALE^1^ (mean score = 67.33, SD = 11.09). One participant (No.22) was excluded due to data collection errors, and the other seven participants were also excluded due to low accuracy (< 70%). The final sample included 44 participants (14 males, mean age, 27.69, SD = 3.78). Their mean age of starting L2 learning was 9.8 years old and the average acquisition time for L2 was 17.89 years. The result of their LexTALE was 68.28 (SD = 11.5).

### 2.2. Materials

In bilingual research on word processing, although adopting translation equivalents is a common practice (i.e., Kazanas and Altarriba, 2016a), we compiled two separate sets of stimuli of each language since participants may show unconscious and nonselective access to words in L1 when they are undergoing a task exclusively in L2 (Wu and Thierry, 2012; see also one behavioral study: Bromberek-Dyzman et al., 2021). In this way, uncontrolled lexico-semantic priming could be avoided.

Given that there are no published normative studies on emotion word type, stimuli in both languages in this study were obtained through a yes/no voting method employed in prior studies (Wang et al., 2019; Liu et al., 2022a,b). In terms of English stimuli, 296 English adjectives whose valence ratings were below 3.5, ranged from 4.0 to 5.0 or above 5.5 on a 9-point Likert scale (1 = very unpleasant, 9 = very pleasant) were selected from an English affective norm database (Warriner et al., 2013) to form a word pool. Then, 20 participants were recruited to classify these selected words according to the definitions of emotion-label, emotion-laden and neutral words. In light of the standard that at least about 80% of participants voted for a specific word type, 83 emotion-label words, 82 emotion-laden words, and 75 neutral words were obtained. Secondly, we recruited three groups of 20 participants to evaluate the familiarity, concreteness, and valence, respectively, and a group of 21 participants to evaluate the arousal of these 240 words selected from the first step on a 7-point Likert scale (7 being very familiar, very abstract, very pleasant, very excited, respectively). All invited raters did not overlap with the samples of the experiment.

With respect to Chinese experimental stimuli, we adhered to the criteria used to select English materials. Firstly, a word pool of 408 words mostly taken from SUBTLEXCH (Cai and Brysbaert, 2010) and partly from prior studies on emotion word type (Zhang et al., 2017; Wang et al., 2019) was created. Then, 20 participants were invited to classify these 408 Chinese words into emotion-label, emotion-laden and neutral words according to their definitions, from which we obtained 101 emotion-label, 94 emotion-laden, and 81 neutral words. Next, we recruited four groups of 20 participants to evaluate these 276 words based on their familiarity, concreteness, valence and arousal, respectively, on the same 7-point Likert scale. All raters did not participate in the experiment.

Finally, 180 adjectives were selected, including 60 emotion-label words (30 positives, 30 negatives), 60 emotion-laden words (30 positives, 30 negatives), and 60 neutral words in Chinese and English. Five groups of words were matched on length/strokes, familiarity, and concreteness in each language (*ps* > 0.1). In both Chinese and English, valence ratings significantly decreased from positive words to neutral words to negative words (*ps* < 0.001). However, in each language, valence for emotion-label and emotion-laden words in each category did not differ (*ps* > 0.15). For the arousal rating of Chinese and English words, four groups of emotion words with different valence were rated significantly higher than neutral words (*ps* < 0.001). Meanwhile, there were no significant differences among them in each language (*ps* > 0.1) [see Table 1 on the Chinese and Table 2 on the English stimulus attributes, respectively].

**Table 1:** Means (M) and standard deviation (SD) for Chinese emotion-label, emotion-laden, and neutral words.

	Positive words		Negative words		Neutral words	Label	Laden	Label	Laden
Valence	5.7 (1.12)	5.8 (1.04)	2.4 (0.96)	2.3 (1.14)	4.2 (0.76)
Familiarity	6.5 (1.16)	6.5 (1.17)	6.5 (1.08)	6.3 (1.34)	6.5 (1.05)
Arousal	5.6 (1.21)	5.4 (1.11)	5.6 (1.04)	5.5 (1.15)	3.03 (1.61)
Concreteness	4.3 (1.27)	4.4 (1.60)	4.2 (1.31)	4.2 (1.55)	4.2 (1.95)
Length	17.6 (4.40)	18.3 (4.73)	17.6 (3.24)	17.7 (3.49)	17.7 (3.76)

**Table 2:** Means (*M*) and standard deviation (SD) for English emotion-label, emotion-laden, and neutral words.

	Positive words		Negative words		Neutral words	Label	Laden	Label	Laden
Valence	5.5 (1.17)	5.5 (1.16)	2.6 (1.28)	2.5 (1.27)	4.1 (0.78)
Familiarity	6.6 (0.96)	6.7 (0.71)	6.6 (0.89)	6.6 (0.94)	6.6 (0.83)
Arousal	5.3 (1.26)	5.2 (1.24)	5.4 (1.21)	5.2 (1.31)	3.1 (1.52)
Concreteness	5.2 (0.98)	5.2 (1.18)	5.2 (0.95)	5.1 (1.08)	5.2 (1.39)
Length	7.5 (2.00)	7.7 (1.54)	7.5 (1.93)	7.5 (2.22)	7.5 (1.57)

### 2.3. Procedure

Participants were tested in a quiet and dimly illuminated room. Experimental stimuli were presented in white on a gray background employing the psychological software PsychoPy (Peirce et al., 2019). This experiment consisted of two language blocks, Chinese and English, presented in random order. Chinese words were presented in Song font, size 24, and English words in Times New Roman font, size 24. In each language block, three unrepeated experiment blocks containing 60 trials (10 words for each emotion word category and 20 neutral words) were presented fully randomized. Prior to the experiment, there was a practice session with 24 trials (12 words in Chinese and 12 words in English) to familiarize participants with the procedure. Each trial started with a fixation “+” in white lasting 250 ms, followed by a blank screen varying between 300 and 500 ms. Subsequently, the stimuli word presented for a maximum duration of 3,000 ms at the center of the screen and would disappear immediately after a response was given. In this experiment, participants were instructed to judge whether a given word was positive, negative, or neutral as quickly and accurately as possible by pressing designated keys counterbalanced across participants. After the experiment, participants were asked to complete valence ratings for the experimental stimuli on a 7-point Likert scale (1 = very unpleasant, 7 = very pleasant).

### 2.4. Statistical modeling

The RTs and ACCs were analyzed by Linear Mixed Effect Models (LMEMs) (Baayen et al., 2008) and Generalized LMEMs (Lo and Andrews, 2015) respectively in R (Version 4.2.1; R Core Team, 2022), using the *lmer4* package (Bates et al., 2015). A box-cox power transformation of response latency (Osborne, 2010) was carried out since such transformation showed better performance in promoting the normality of the errors than log-transformed RTs and raw RTs, which was dependent on the residual sum of squares of our basic model. Participants and items were treated as random intercepts, allowing us to estimate how much variability in the random group factors of participants and items. Five groups of words (positive emotion-label words, positive emotion-laden words, negative emotion-label words, negative emotion-laden words, and neutral words) were treated as fixed effects. We adopted *hypr* package (Rabe et al., 2020) to design a repeated contrast coding to compare each type of emotion stimuli to neutral ones, and the same model was applied in Chinese and English, respectively. Given that random factors are the sources of stochastic variability (Barr et al., 2013), the ‘maximal model’ included all relevant random structures. Following the suggestions of Barr et al. (2013), we first removed the correlations among the random factors and then interactions when the full model did not reliably converge. To make sure the estimation did not end prematurely, all final models successfully converged after a restart with the appropriate choice of optimizers (using *lergotrile*). The resulting models were compared to the model with maximal random structure using the chi-square difference test and Akaike’s Information Criterion, for which a lower value indicates better model fit (Kline, 2011). Notably, for the model trimming procedure, the final models and their corresponding maximal models did not diverge in their results, suggesting that a parsimonious and interpretable model could provide the best fit to the data.

A three-step approach was applied to code fixed terms due to the unbalanced nature of the current experiment. In Model 1, repeated contrasts were specified to compare and collapse the four groups of emotion words to the neutral words within each language. This allowed us to investigate the cost of emotion-label or emotion-laden words with positive or negative valence, examining the facilitation or interference elicited by emotion word type or valence. Model 2 excluded the neutral condition and sum-contrast coding was defined, in a way that the intercept of the model represented the grand mean value of the fixed factors (Schad et al., 2020). This model enabled us to directly examine the main effect of and interaction between emotion word type (emotion-label vs. emotion-laden), valence (positive vs. negative), and language (Chinese vs. English). The *emmeans* package (Lenth, 2017) was used to conduct post-hoc analysis in this model and first determine the estimated marginal means (EMMs) and their standard errors, and then make the pair-wise comparisons. To prevent *emmeans* from calculating the df for the EMMs, we applied asymptotic dfs (i.e., *z* values and tests). In Model 3, we split the data into a corresponding subset of English words only and included L2 proficiency as a fixed factor. Then, we computed the statistical models again for valence, emotion word type and proficiency to detect the modulation effect of L2 proficiency on emotion word processing.

## 3. Results

Of the overall 18,720 data points, data from one participant (Subject No.22) were eliminated due to his/her failure to activate the response key. After applying a threshold of <70% correct response as exclusion criteria, data from the other seven participants, as well as a total of nine items presented either in Chinese (“紧急” means “urgent,” “明确” means “clear,” “准时” means “punctual,” “冷静” means “calm”) or in English (“concerned,” “contented,” “moved,” “sympathetic,” “thrilled”) blocks were excluded, leaving a total of 44 participants and 351 stimuli for further accuracy analysis (15,307 trials). For the analysis of response latency, wrong (overall, *n* = 952, 6% of trials) and missing (overall, *n* = 181, 0.1%) responses were coded as errors and were discarded. We applied model criticism (see Baayen, 2008; Baayen and Milin, 2010) to remove data points (*n* = 477, 3%) that deviated more than a range of −2.5 to 2.5 standardized residual errors. The models were afterward re-fitted on the truncated dataset with a total of 13,878 trials. The average RTs and ACCs across conditions are displayed in Table 3.

**Table 3:** Mean RTs (ms) and ACCs (%) of five groups of words in L1 (Chinese) and L2 (English).

	RTs (response times)		ACCs (accuracy rates)	
	Chinese	English	Chinese	English
Positive emotion-label	815 (264)	1,029 (346)	97.3 (16.4)	92.5 (26.4)
Positive emotion-laden	849 (269)	1,053 (316)	94.7 (22.3)	93.5 (24.7)
Negative emotion-label	886 (286)	1,098 (333)	96.6 (18.2)	92.6 (26.1)
Negative emotion-laden	895 (290)	1,158 (353)	96.4 (18.7)	88.5 (31.9)
Neutral	971 (309)	1,187 (370)	92.4 (26.6)	94.2 (23.3)

Firstly, to investigate the emotion effects elicited by emotion words, the comparison between neutral words and the four groups of emotion words reflected in RTs and ACCs in both L1 and L2 was made (see Table 4). For RTs, the results revealed that in L1, compared to neutral words, both types of emotion words were responded to significantly faster. In L2, while negative emotion-label words, positive emotion-label words, and positive emotion-laden words showed significant higher processing speed than neutral words, there was no difference between negative emotion-laden words and neutral words. For ACCs, it showed that L1 negative emotion-label words, negative emotion-laden words, and positive emotion-label words were responded to more accurately as compared to neutral words. However, the difference between positive emotion-laden and neutral words in L1 did not reach significance. In L2, negative emotion-label words, positive emotion-label words, and positive emotion-laden words had similar ACCs to neutral words. Nevertheless, it was found that more errors were made with negative emotion-laden words than neutral words.

**Table 4:** Statistical analysis of the RTs and ACCs of four groups of emotion words compared to neutral words in L1 (Chinese) and L2 (English).

	RTs (response times)				ACCs (accuracy rates)				*β*	SE	*t*	*p*	*β*	SE	z	*p*
Chinese	Positive emotion-label vs. Neutral	−0.005	0.00052	−9.6	<0.0001***	1.1	0.31	3.7	0.00025***
	Positive emotion- laden vs. Neutral	−0.0037	0.00052	−7.2	<0.0001***	0.49	0.28	1.8	0.08
	Negative emotion-label vs. Neutral	−0.0027	0.00052	−5.2	<0.0001***	0.79	0.29	2.7	0.0064**
	Negative emotion-laden vs. Neutral	−0.0024	0.00052	−4.5	<0.0001***	0.85	0.3	2.9	0.0043**
English	Positive emotion-label vs. Neutral	−0.0038	0.00082	−4.7	<0.0001***	−0.25	0.28	−0.88	0.38
	Positive emotion-laden vs. Neutral	−0.0029	0.00077	−3.8	0.00019***	−0.12	0.27	−0.47	0.64
	Negative emotion-label vs. Neutral	−0.0018	0.00077	−2.3	0.024*	−0.24	0.26	−0.91	0.36
	Negative emotion-laden vs. Neutral	−0.00053	0.00077	−0.69	0.49	−0.86	0.25	−3.4	0.00074***

*p < 0.05, **p < 0.01, ***p < 0.001.

Secondly, the RTs and ACCs of the four groups of emotion words in L1 and L2 were compared (see Table 5). For RTs, the main effects of language, valence, and emotion word type were observed. Specifically, Chinese-English bilinguals responded faster to Chinese words relative to English words, to positive words relative to negative words, and to emotion-label words relative to emotion-laden words. No interactions between emotion word type, valence and language were observed in reaction times.

**Table 5:** Statistical analysis of the main effects of and interaction between valence, emotion word type and language reflected in RTs and/or ACCs among the four groups of emotion words in L1 (Chinese) and L2 (English).

	RTs (response times)				ACCs (accuracy rates)			
	*β*	SE	*t*	*p*	*β*	SE	z	*p*
Valence	0.001	0.00021	4.9	<0.0001***	−0.18	0.19	−0.99	0.32
Emotion word type	−0.00048	0.0002	−2.3	0.02*	−0.26	0.18	−1.4	0.15
language	−0.0033	0.00028	−12	<0.0001***	−0.92	0.22	−4.1	<0.0001***
Valence × Emotion word type	8.7e-05	0.00019	0.44	0.66	−0.022	0.34	−0.065	0.95
Valence × Language	−9.4e-05	0.00019	−0.48	0.63	−0.36	0.35	−1	0.3
Emotion word type × Language	6.8e-05	0.00019	0.35	0.73	0.0076	0.35	0.22	0.83
Valence × Emotion word type × Language	0.00016	0.00019	0.84	0.4	−1.5	0.68	−2.2	0.03*

*p < 0.05, **p < 0.01, ***p < 0.001.

**Figure 1:**
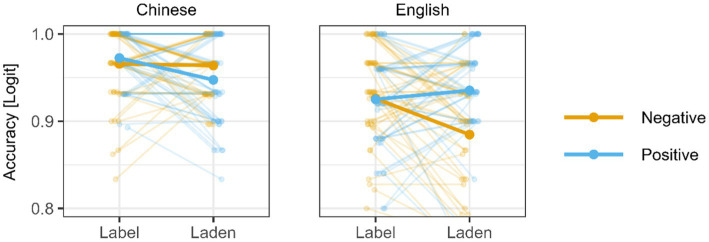
Triple interaction between valence (in color), language (L1 on the left, L2 right), and emotion word type (emotion-label and emotion-laden; on the x axis) reflected in ACCs (on the y axis). Single-subject indices (thin lines) are overlayed by group averages (thick lines).

ACCs revealed a main effect of language, with Chinese words being responded to more accurately than English words. Moreover, the emotion word type × valence × language interaction was significant (see Figure 1). *Post hoc* analysis showed (1) in English, Chinese-English bilinguals responded significantly more accurately to positive emotion-laden words than negative emotion-laden words (*β* = 0.7415, SE = 0.320, *z* = 2.316, *p* = 0.0206), as well as marginally more accurately to negative emotion-label words than negative emotion-laden words (*β* = 0.594, SE = 0.312, *z* = 2.316, *p* = 0.0570). No such differences were found in Chinese. (2) Chinese positive emotion-label words (*β* = 1.147, SE = 0.397, *z* = 2.887, *p* = 0.0039), negative emotion-label words (*β* = 0.774, SE = 0.363, *z* = 2.131, *p* = 0.0331), and negative emotion-laden words were responded more accurately than those in English (*β* = 1.430, SE = 0.362, *z* = 3.956, *p* = 0.0001). However, there was no difference between positive emotion-laden words in Chinese and English (*β* = 0.340, SE = 0.363, *z* = 0.936, *p* = 0.3492).

In terms of the role of L2 proficiency, the effect of L2 proficiency was significant in English word processing (*β* = −0.0012, SE = 0.00039, *t* = −3, *p* = 0.0046), suggesting the higher the English proficiency, the faster the reaction times. However, it revealed null effects of L2 proficiency on the language effect, valence effect, emotion word type effect, and their interactions (*ps* > 0.66) observed in L2.

The correlation coefficient between two variables is expected to have a minimum value of 0.2 to be practically significant. In the present study, the subjective valence ratings for all the experimental stimuli after the experiment showed a positive correlation with the established reference values of valence (Chinese: *r* = 0.56, Crl [0.36, 0.74]; English: *r* = 0.44, Crl [0.21, 0.64]).

## 4. Discussion

In the present study, we employed an ECT to examine whether emotion adjectives that straightforwardly label affective states (emotion-label words) and trigger emotions (emotion-laden words) are embodied in Chinese-English speakers, with Chinese as their dominant (L1) and English as the non-dominant language (L2). In the following parts, we discussed the embodiment of these two kinds of emotion words from two aspects, the emotion effect and the emotion word type effect in both L1 and L2, to further clarify how the two sorts of emotion words are embodied and processed differently from neutral stimuli and from each other.

### 4.1. The emotion effect of emotion-label and emotion-laden words in L1 and L2

As we mentioned in the Introduction, the emotion effect refers to the processing facilitation for emotion words relative to neutral words. In the present study, the emotion effect was observed for these two types of emotion words in L1. To be specific, facilitation was found for emotional stimuli, regardless of their emotion word type and valence, in comparison to neutral ones, corresponding to faster RTs. This disadvantage for neutral words relative to emotion words observed in past studies (e.g., Kousta et al., 2009; Goh et al., 2016) was replicated here, indicating that L1 emotion words, either explicitly label affective states or evoke emotions through words’ connotations could activate emotions which speeds up the clarification. As for the ACCs, some researchers argued that the analysis of accuracy was inappropriate in some cognitive tasks in which the emotional dimension is task-relevant, such as the ECT and affective decision task (Ferré et al., 2018; Liao and Ni, 2021). The reason lies in the fact that valence categorization may involve subjective experience, leading participants to classify some words in a category that differs from the referenced one (González-Villar et al., 2014). However, such responses should not be regarded as categorization errors. In an attempt to rule out this confounding possibility, participants in the present study were required to rate the valence of stimuli after the experiment, and the result showed a positive correlation between the offline rating and the established reference values of valence. Therefore, ACCs were analyzed in this ECT task and responses that did not match the reference valence values were considered incorrect. Finally, the analysis of accuracy revealed that the emotion effect found in RTs was reflected in ACCs except for positive emotion-laden words. That is, while positively valenced emotion-label words and both types of negative emotion words were categorized more accurately than neutral words in L1, no significant difference was observed in ACCs between positive emotion-laden and neutral words. A possible interpretation might be that emotion-laden words have indirect semantic associations with emotions (Knickerbocker and Altarriba, 2013), as one emotion-laden word corresponds to multiple connections with the general lexicon. For example, the emotion-laden adjective “successful” may evoke emotions like “happy” or “excited.” In this sense, we think that such ambiguous associations with emotion concepts increase the difficulty in categorizing the valence of emotion-laden words. Furthermore, the positive effect may broaden the scope of attention, impair cognitive performance and widen associations with words resulting in more diffuse semantic activation (Phillips et al., 2002). This is exactly the case for the performance of healthy participants in this study. Therefore, no emotion effect of positive emotion-laden words was observed in ACCs.

In English, results from the RTs indicated that the emotion effect emerged for emotion words except for negative emotion-laden words. Specifically, negative emotion-label words and both types of positive emotion words were processed with significant shorter RTs, whereas no significant difference in processing negative emotion-laden words and neutral words was observed (negative emotion-laden words did not differ from neutral words). This finding suggested that negative emotion-laden words in English, the non-dominant language, activated similar emotion with neutral words. One possible reason for this finding is that emotion-laden words bear no direct connection to their affective meanings, and thus are not well grounded in emotional experiences, resulting in less or weak emotion activation in L2, the less emotionally embodied language (Caldwell-Harris, 2014). Additionally, emotion-laden words may be particularly susceptible to the negative valence in L2, leading to narrow and enhanced selective attention effect (Finucane, 2011), which in turn slows down responses. This finding found support in one previous ERPs study (Zhang et al., 2019a) which investigated how L2 emotion-label and emotion-laden words affected conflict processing in a flanker task. It was found that compared to the incongruent condition, enhanced left frontal N200 was elicited by merely L2 negative emotion-label words in the congruent condition. Nevertheless, negative emotion-laden and neutral stimuli did not shape N200. Taken together, the finding in this study allowed us to speculate that L2 negative emotion-laden words might be disembodied.

This finding in L2 was also reflected in the accuracy data, showing that negative emotion-laden words had the lowest ACCs among the four types of emotion words and neutral words. To be specific, both kinds of positive emotion words and negative emotion-label ones were classified as accurately as neutral words, whereas less accurate responses were made to negative emotion-laden words than neutral words. A possible reason for this finding was that the L2 experimental stimuli used in this study were quite familiar to participants, contributing to the ceiling effect. However, it needs to be aware that the lower ACCs of L2 negative emotion-laden words, together with their longer RTs, jointly indicate that they are disembodied. Prior studies, which unsystematically mixed the two sorts of emotion words, have controversial results about whether emotion words in L2 are disembodied (Kühne and Gianelli, 2019). Our results showed that the embodiment of L2 emotion words is modulated by the emotion word type (C. Wu and Zhang, 2019), thereby shedding light on the extant conflicting results concerning the embodiment in L2, at least for negative emotion-laden words.

### 4.2. The emotion word type effect on emotion word processing in L1 and L2

In this section, we discuss the effect of emotion word type effect, that is, how emotion-label and emotion-laden words are processed differently in both L1 and L2, as well as the modulation of valence on this effect. The results revealed several main effects. Firstly, a main effect of language demonstrated that participants showed slower and less accurate responses to emotion words in English compared to Chinese, suggesting that they were more proficient in L1. This finding is consistent with the language profile of participants who live in their L1 environment and are dominant in their L1 (Chen et al., 2015). As far as the role of L2 language proficiency is concerned, our finding showed that only RTs to L2 words were modulated by English proficiency, with faster responses observed among participants with higher levels of English proficiency. This finding is in line with the idea that increasing L2 proficiency strengthens the connection between L2 words’ forms and their conceptual meanings (Kroll et al., 2010). Therefore, in the present study, late Chinese-English bilinguals who acquired L2 *via* instructional settings (e.g., school or class) responded more quickly to English words as their English proficiency improved.

In addition, it was found that Chinese–English bilinguals were slower in responding to negatively valenced words when compared to positively valenced words in both L1 and L2. This finding is consistent with some relevant research on the disassociation between emotion-label and emotion-laden words. For example, the behavioral data from a recent ERPs study (Liu et al., 2022b) demonstrated that negative words produced longer reaction times than positive words in both the ECT and emotional Stroop tasks. Similar effects were observed in a priming LDT (Kazanas and Altarriba, 2016a), which confirmed that emotion words with positive information enhance word processing across languages (Spanish and English). This superiority effect for positive words with faster performance is in line with the positivity bias (Hofmann et al., 2009). Two possible explanations have been proposed for this phenomenon. On the one hand, the density hypothesis suggests that positive words are more densely clustered and connected in memory than negative words, which results in the processing advantage for positive words (Unkelbach et al., 2008). On the other hand, from a survival perspective, negative stimuli can lead to a cognitive “freezing” when individuals are presented with negative or threatening information (Algom et al., 2004). As mentioned in the Introduction part, so far, the valence effect is still a controversial matter (Kauschke et al., 2019). Nevertheless, our study, together with other emotion word type research (e.g., Kazanas and Altarriba, 2016a; Bromberek-Dyzman et al., 2021), confirms the presence of a positivity bias in emotion word processing even after we systematically categorized emotion-label and emotion-laden words.

The most relevant finding of this experiment is the observation of the processing facilitation for emotion-label over emotion-laden words in both L1 and L2. Consistent with our hypothesis, Chinese-English bilinguals in this study tended to take a shorter time to respond to emotion-label words, regardless of language operation, even after controlling the concreteness and the word class of stimuli. This finding stands in contrast to a study by Bromberek-Dyzman et al. (2021), as well as the behavioral data reported in a previous ERPs study (Zhang et al., 2019b) which reported a processing advantage for emotion-laden words relative to emotion-label words, with higher processing speed and accuracy. However, our finding corroborated a series of behavioral studies (Knickerbocker and Altarriba, 2013; Kazanas and Altarriba, 2015, 2016a,b), as well as the behavioral data in a recent ERPs study (Liu et al., 2022a) which reported facilitated processing of emotion-label rather than emotion-laden words.

This finding showed that although both emotion-label and emotion-laden words eventually activate emotions in the ECT, these two types of emotion words are processed differently, providing evidence for the emotion word type effect. Such an effect could be explained from several possible explanations. One possible account is the “mediated account” (Altarriba and Basnight-Brown, 2011; Wu et al., 2021a) which suggests that emotion-laden words could be viewed as a kind of “mediated” affective concepts. Thus, their emotional meanings could be accessed only through a “mediated event” that links the conceptual meanings and associated affective experiences. On the contrary, emotion-label words explicitly label emotions, making it easier to automatically or unconsciously approach their affective components. Another possible explanation is the emotion duality model, which may shed light on the distinction between the two kinds of emotion words. Accordingly, emotions activated by emotion-label words are more automatic and biologically rooted, while emotions induced by emotion-laden words are thought to be based on a reflective system that needs more cognitive effort (Imbir et al., 2019; Liu et al., 2022b). Furthermore, from an embodied cognition perspective, emotion-label words are more strongly shaped in socialization and emotional interaction as they directly denote a specific emotional state (e.g., feeling happy or sad) (Liu et al., 2022a). It has also been shown that emotion-label words are acquired at an earlier age and are more attached to life experiences than emotion-laden words (Basnight-Brown and Altarriba, 2018). Therefore, it is conceivable that RTs to emotion-laden words were longer than those to emotion-label words. Another intriguing finding was the absence of an interaction between valence and emotion word type in RTs. However, a triple interaction between the emotion word type, valence and language was observed in ACCs. Specifically, Chinese-English bilinguals responded less accurately to negative emotion-laden words as compared to positive emotion-laden words and negative emotion-label words in English only. Given the participants in this study are native Chinese speakers, it is plausible to infer that they are able to ground emotion words in their emotional experiences so that equally fewer errors were made in categorizing the valence of stimuli in L1. However, L2 negative emotion-laden words are probably disembodied, resulting in more categorization errors relative to both L2 positive emotion-laden words and negative emotion-label words. Sheikh and Titone (2016) found in a previous study that the emotional embodiment might be absent for negative words but not positive words in L2. In the present study, we extended their findings by illustrating that it is L2 negative emotion-laden words but not L2 negative emotion-label words that are likely to be at risk of emotional disembodiment. It is also of importance to note that emotion-label words and negative emotion-laden words in Chinese were responded to more accurately than in English, while no difference was found in processing positive emotion-laden words between Chinese and English. The poor performance for positive emotion-laden words relative to the other three groups of words in L1 lent support to the claim that the processing difference between these two types of emotion words may be more robust in L1 positive words than in negative ones (Kazanas and Altarriba, 2015, 2016a; Wang et al., 2019), which still needs future studies to verify it.

### 4.3. Limitation and future direction

In this study, while the stimulus attributes were matched in each language, they did not match between L1 and L2. However, this does not impact our main findings, as the emotion effect and the effects of valence and emotion word type were observed across languages. Future research may benefit from strict control on stimuli between languages to make the results more comparable. In addition, it is urgent to conduct normative studies that distinguish emotion-label from emotion-laden words in Chinese or other languages. To our knowledge, currently, there is only one normative study (Pérez-Sánchez et al., 2021) that provides a set of 1,286 emotion words in the Spanish language using the prototypical approach. Accordingly, the higher the prototypicality of an emotion word is, the more likely it is to be defined as an emotion-label word. Furthermore, the direct comparison of emotion word type effect in L1 and L2 calls for future research using different neuroimaging techniques, such as the electroencephalogram and functional magnetic resonance imaging. Lastly, it has been pointed out that languages conceptualize emotions divergently (Bromberek-Dyzman et al., 2021), and culture is involved in the way individuals store and process emotional information (Basnight-Brown and Altarriba, 2018). Therefore, cross-cultural and cross-linguistic studies are needed to investigate how these two types of emotion words, directly or indirectly related to emotions are processed among individuals with different cultural and linguistic backgrounds.

## 5. Conclusion

In the present study, we aimed to explore the extent to which emotion-label and emotion-laden words are embodied in L1 and L2 among Chinese-English bilinguals. Our results showed that while the emotion effect was absent for L2 negative emotion-laden words, the other types of emotion words, either explicitly refer to emotions or evoke emotional states indirectly, have a processing advantage (decreased RTs and/or higher categorization ACCs) over neutral words in both L1 and L2. Of particular importance, processing facilitation for emotion-label rather than emotion-laden words was found in both languages. In addition, it seems that negative emotion-laden words are responded to less accurately than positive emotion-laden words, as well as negative emotion-label words in L2 only. Altogether, the results indicated the disembodiment of L2 negative emotion-laden words and evidenced the disassociation between the two types of emotion words across languages. These findings provide new insights into the embodiment of emotion words in bilinguals and highlight the importance of considering the role of emotion word type in the context of emotion word research.

## Data availability statement

The raw data supporting the conclusions of this article will be made available by the authors, without undue reservation.

## Ethics statement

The studies involving human participants were reviewed and approved by the Research Ethics Committee of Dalian University of Technology. The patients/participants provided their written informed consent to participate in this study.

## Author contributions

DT, HW, and TK contributed to the experimental design and data collection. YF and DT analyzed the data. DT wrote the manuscript. HW and TK offered suggestions on the writing of the manuscript. DT, YF, HW, BL, AZ, and TK revised the manuscript. All authors contributed to the article and approved the submitted version.

## Funding

This work was supported by the scholarship from China Scholarship Council (No. 201906060171).

## Conflict of interest

The authors declare that the research was conducted in the absence of any commercial or financial relationships that could be construed as a potential conflict of interest.

## Publisher’s note

All claims expressed in this article are solely those of the authors and do not necessarily represent those of their affiliated organizations, or those of the publisher, the editors and the reviewers. Any product that may be evaluated in this article, or claim that may be made by its manufacturer, is not guaranteed or endorsed by the publisher.

## Footnotes

^1^ https://www.lextale.com/
