# Supplementary material for: The embodiment of emotion-label words and emotion-laden words: Evidence from late Chinese–English bilinguals
Source: Front Psychol. 2023 Mar 22;14:1143064. doi: 10.3389/fpsyg.2023.1143064 (PMC10074490; doi:10.3389/fpsyg.2023.1143064)
Supplement: Supplementary file 1 [file Data_Sheet_1.docx]

***Supplementary Material***

**Supplementary 1.** Chinese experimental material

| Positive emotion-label words | Positive emotion-laden words | Negative  emotion-label words | Negative  emotion-laden words | Neutral words |
| --- | --- | --- | --- | --- |
| 愉悦  快乐  兴奋  愉快  喜悦  欣喜  欢乐  欢喜  开心  高兴  舒心  喜乐  欢愉  欣慰  激奋  欢快  激动  畅快  亢奋  欢欣  欣悦  快活  欢畅  痛快  得意  惊喜  冷静  自豪  顺心  满意 | 热闹  浪漫  高雅  慈祥  如意  优秀  亲热  壮观  亲密  圣洁  成功  勇猛  顽强  辉煌  精彩  卓越  吉利  雄伟  壮丽  兴旺  漂亮  高尚  旺盛  阳刚  富足  友好  灿烂  温暖  光荣  繁荣 | 惆怅  害怕  懊恼  愤怒  悲伤  悲愤  难过  焦急  惶恐  悲哀  哀愁  慌张  厌烦  哀伤  内疚  郁闷  沮丧  忧愁  悲切  伤感  失落  忧虑  不安  忧郁  抑郁  失望  苦恼  紧张  委屈  苦闷 | 险恶  阴险  野蛮  放荡  奸诈  恶劣  荒唐  猖狂  虚荣  堕落  低贱  危急  自私  吝啬  荒凉  狼狈  粗鲁  懦弱  狡诈  疼痛  残忍  肮脏  低劣  狡猾  衰老  虚伪  紧急  凶狠  偏执  扭曲 | 系统  标准  适量  简短  平衡  额外  相同  典型  具体  日常  寻常  根本  偶然  综合  所有  必要  基本  横向  分明  分散  间接  明显  一致  业余  短暂  同等  抽象  规范  微观  初步  隐约  轻微  简单  正常  完整  直观  合法  共同  普通  直接  通俗  全面  固定  普遍  平均  实际  任意  特别  规矩  客观  相符  模糊  足够  明确  细微  平常  准时  传统  适当  熟悉 |

**Supplementary 2.** English experimental material

| Positive emotion-label words | Positive emotion-laden words | Negative  emotion-label words | Negative  emotion-laden words | Neutral words |
| --- | --- | --- | --- | --- |
| delighted  happy  excited  cheerful  joyful  pleased  thrilled  joyous  surprised  grateful  pleasant  glad  cheery  moved  gladsome  calm  contented  satisﬁed  relaxed  enjoyable  relieved  sympathetic  entertained  thankful  fond  proud  passionate  concerned  fulfilled  optimistic | elegant  fruitful  brave  prosperous  beautiful  tasty  romantic  diligent  brilliant  wealthy  excellent  glorious  creative  handsome  clever  delicious  graceful  bright  magical  famous  honorable  rich  harmonious  precious  worthy  youthful  energetic  humorous  supportive  perfect | depressed  sad  grieved  ashamed  unhappy  upset  disappointed  afraid  sorrowful  panic  scared  terriﬁed  fearful  nervous  frightened  worried  bored  regretful  shocked  disgusted  frustrated  jealous  guilty  desperate  stressed  awkward  dreadful  pessimistic  envious  lonely | foolish  disastrous  divorced  dangerous  bloody  lazy  stupid  selfish  difficult  poor  catastrophic  worthless  greedy  criminal  failed  illegal  destructive  arrogant  poisonous  rude  unfortunate  ridiculous  violent  tough  ignorant  harmful  cruel  tragic  aggressive  deadly | countless  annual  occasional  behavioral  current  various  verbal  obvious  numerous  topical  probable  usual  global  neutral  apparent  visual  common  same  similar  concrete  extra  public  parallel  direct  average  frequent  actual  additional  straight  automatic  slight  regional  central  immediate  formal  biological  theoretical  external  manual  initial  genetic  foreign  eventual  temporary  identical  continuous  speciﬁc  systematic  local  daily  general  national  mechanical  absolute  collective  worldly  personal  private  objective  previous |
