# Supplementary material for: The embodiment of emotion-label words and emotion-laden words: Evidence from late Chinese–English bilinguals
Source: Front Psychol. 2023 Mar 22;14:1143064. doi: 10.3389/fpsyg.2023.1143064 (PMC10074490; doi:10.3389/fpsyg.2023.1143064)
Supplement: Supplementary file 3 [file Data_Sheet_3.docx]

# Load libraries of functions required for analyses.

library(tidyverse);library(nlme);library(readxl);library(ez);library(lattice)

library(lme4);library(dplyr);library(lattice);library(latticeExtra);library(lmerTest)

library(ggplot2)

library(MASS)

library(car)

library(emmeans)

library(PairedData)

library(hypr)

library(lmerOut)

# -- Get information on package versions.

sessionInfo()

# data arrangement

dat_emotion <- read.csv("50data0914.csv", stringsAsFactors=FALSE)

str(dat_emotion)

#critical variable set as factors

dat_emotion <- do.call(data.frame, dat_emotion)

dat_emotion$condition <- as.factor(dat_emotion$condition)

dat_emotion$participant <- as.factor(dat_emotion$participant)

dat_emotion$item <- as.factor(dat_emotion$item)

dat_emotion$language <- as.factor(dat_emotion$language)

#obtain accuracy and participant removal

dat_emotion<- dat_emotion %>%

filter(key_CF1.keys != "None" & key_CF1.rt != "NA")

#dat_emotion$key_CF1.keys <- droplevels(dat_emotion$key_CF1.keys)

dat_emotion$Accuracy <- ifelse ((dat_emotion$answer == dat_emotion$key_CF1.keys),1, 0)

dat_emotion<- dat_emotion %>%

filter(participant != "22")

dat_emotion$participant <- droplevels(dat_emotion$participant)

#Join proficiency

dat_proficiency <- read_csv("English Proficiency.csv") %>%

mutate(participant = as.character(participant)) %>%

filter(participant != "22") %>%

mutate(proficiency_std = (grade - mean(grade)) / sd(grade))

str(dat_proficiency)

dat_emotion <- left_join(dat_emotion, dat_proficiency, by = "participant")

#errors per condition

errors <- aggregate(Accuracy ~ condition + language + participant, dat_emotion, mean)

view(errors)

high_errors <- errors[errors$Accuracy < .7,]$participant

high_errors <- unique(high_errors)

## remove high-error participants

dat_emotion <- dat_emotion[!(dat_emotion$participant %in% high_errors),]

length(table(dat_emotion$participant))

#errors per item

errors1 <- aggregate(Accuracy ~ item, dat_emotion, mean)

sort(errors1[,2])

error_items <- errors1[errors1$Accuracy< .65,]$item

dat_emotion <- dat_emotion[!(dat_emotion$item %in% error_items),]

str(dat_emotion)

# exclude error trials

table(dat_emotion$Accuracy)

table(dat_emotion$Accuracy)["0"]/length(dat_emotion$Accuracy)

dat_emotion_RT <- dat_emotion[dat_emotion$Accuracy == 1,]

dat_emotion_RT <- na.omit(dat_emotion_RT)

nrow(dat_emotion_RT)/nrow(dat_emotion)

##### outlier removal #####

str(dat_emotion_RT)

dat_emotion_RT$RTms <- dat_emotion_RT$key_CF1.rt*1000

romr.fnc <-

function(model,dat_emotion_RT, trim = 2.5){

dat_emotion_RT$rstand = as.vector(scale(resid(model)))

row.names(dat_emotion_RT)=1:nrow(dat_emotion_RT)

outliers=as.numeric(row.names(dat_emotion_RT[abs(dat_emotion_RT$rstand)>trim,]))

data0=dat_emotion_RT

if(length(outliers) > 0){

dat_emotion_RT=dat_emotion_RT[-outliers,,drop=TRUE]

}

cat("n.removed =",(nrow(data0)-nrow(dat_emotion_RT)),"\n")

cat("percent.removed

=",(nrow(data0)-nrow(dat_emotion_RT))/nrow(data0)*100,"\n")

return(list(dat_emotion_RT=dat_emotion_RT,data0=data0,n.removed=nrow(data0)-nrow(dat_emotion_RT),percent.removed=(nrow(data0)-nrow(dat_emotion_RT))/nrow(data0)*100))

}

mod0='RTms ~ condition * language + (1|participant) + (1|item)'

res0=lmer(eval(parse(text=mod0)), data=dat_emotion_RT)

d.romrDATA_dat_emotion=romr.fnc(res0,dat_emotion_RT)$dat_emotion_RT

str(d.romrDATA_dat_emotion)

#########Descriptive###############

library(plyr)

dat_Summary <- ddply(d.romrDATA_dat_emotion, c("condition","language"), summarise,

N = length(RTms),

meanRTs = mean(RTms),

sd = sd(RTms),

se = sd / sqrt(N)

)

dat_Summary

####################

##### Analysis #####

head(d.romrDATA_dat_emotion)

x11() ;truehist(d.romrDATA_dat_emotion$RTms)

# Fist look to a basic model in order to check residuals

mod0='RTms ~ condition * language + (1|participant) + (1|item)'

res0=lmer(eval(parse(text=mod0)), data=d.romrDATA_dat_emotion)

x11(); qqPlot(residuals(res0))

# a clear non normality of residuals.

lambda=car::powerTransform(lmer(eval(parse(text=mod0)), data=d.romrDATA_dat_emotion))

summary(lambda)

lambda

# box-cox power transformation of rt

d.romrDATA_dat_emotion$rts.p=((d.romrDATA_dat_emotion$RTms^-0.5256 - 1)/-0.5256)

x11() ;truehist(d.romrDATA_dat_emotion$rts.p)

mod00='log(RTms) ~ condition * language + (1|participant) + (1|item)'

mod000='rts.p ~ condition * language + (1|participant) + (1|item)'

res00=lmer(eval(parse(text=mod00)), data=d.romrDATA_dat_emotion,

control = lmerControl(optimizer = "bobyqa",

optCtrl = list(maxfun=2e6)))

res000=lmer(eval(parse(text=mod000)), data=d.romrDATA_dat_emotion,

control = lmerControl(optimizer = "bobyqa",

optCtrl = list(maxfun=2e6)))

x11(); qqPlot(residuals(res00))

x11(); qqPlot(residuals(res000))

# residuals better than with log transformation

# First Step (neutral comparison)

str(d.romrDATA_dat_emotion)

#keeping only for each language：Chinese

dat_emotion_Chinese <- subset(d.romrDATA_dat_emotion, language=="Chinese")

dat_emotion_Chinese$language <- droplevels(dat_emotion_Chinese$language)

str(dat_emotion_Chinese)

#repeated contrasts

str(dat_emotion_Chinese)

unique(dat_emotion_Chinese$condition)

HcRep <- hypr(

Negative_labelvsNeutral = negativelb ~ neutral,

Negative_ladenvsNeutral = negativeld ~ neutral,

Positive_labelvsNeutral = positivelb ~ neutral,

Positive_ladenvsNeutral = positiveld ~ neutral,

levels = c("negativelb", "negativeld", "neutral", "positivelb", "positiveld")

)

HcRep

MASS::fractions(MASS::contr.sdif(5))

contrasts(dat_emotion_Chinese$condition) <- contr.hypothesis(HcRep)

#try to maximal

mod_chinese1='rts.p ~ condition + (condition|participant) + (condition|item)'

res.1p.chinese=lmer(eval(parse(text=mod_chinese1)), data=dat_emotion_Chinese,

control = lmerControl(optimizer = "bobyqa",

optCtrl = list(maxfun=2e6)))

summary(res.1p.chinese)$varcor

#sigularfit

summary(res.2p.chinese<- update(res.1p.chinese,.~.-(condition|item)

+(1|item)))

summary(res.3p.chinese<- update(res.2p.chinese,.~.-(condition|participant)

+(1|participant)))

pprint(summary(res.3p.chinese), "Main_lmerFit_Chinese.html", type = "html")

#keeping only for each language：English

dat_emotion_English <- subset(d.romrDATA_dat_emotion, language=="English")

dat_emotion_English$language <- droplevels(dat_emotion_English$language)

str(dat_emotion_English)

#repeated contrasts

contrasts(dat_emotion_English$condition) <- contr.hypothesis(HcRep)

mod_English='rts.p ~ condition + proficiency_std + (1|participant) + (1|item)'

res.1p.English=lmer(eval(parse(text=mod_English)), data=dat_emotion_English,

control = lmerControl(optimizer = "bobyqa",

optCtrl = list(maxfun=2e6)))

summary(res.1p.English)

pprint(summary(res.1p.English), "Main_lmerFit_English.html", type = "html")

# Second Step (remove neural)

dat_emotion_3way <- subset(d.romrDATA_dat_emotion, condition !="neutral")

dat_emotion_3way$condition <- droplevels(dat_emotion_3way$condition)

library(forcats)

dat_emotion_3way$emotion <- fct_collapse(dat_emotion_3way$condition, Negative = c("negativelb","negativeld"), Positive = c("positivelb","positiveld"))

dat_emotion_3way$Label <- fct_collapse(dat_emotion_3way$condition, Label = c("negativelb","positivelb"), Laden = c("negativeld","positiveld"))

str(as.factor(dat_emotion_3way$participant))

View(dat_emotion_3way)

dat_Summary_3way_summary <- ddply(dat_emotion_3way, c("emotion", "Label","language"), summarise,

N = length(RTms),

meanRTs = mean(RTms),

sd = sd(RTms),

se = sd / sqrt(N)

)

dat_Summary_3way_summary

sink('3_way_RTs.txt')

print(dat_Summary_3way_summary)

sink()

#sum contrast coding

getOption("contrasts")

options(contrasts = c("contr.sum","contr.poly"))

#try to maximal

#mod_maximal='rts.p ~ emotion * label * language + (emotion * label * language |participant) + (emotion * label * language|item)'

#res.1p.3way=lmer(eval(parse(text=mod_maximal)), data=dat_emotion_3way,

# control = lmerControl(optimizer = "bobyqa",

# optCtrl = list(maxfun=2e6)))

#summary(res.1p.3way)$varcor

#mod_reduce1='rts.p ~ emotion * Label * language + (emotion * Label * language || participant) + (emotion * Label * language ||item)'

#res.2p.3way=lmer(eval(parse(text=mod_maximal)), data=dat_emotion_3way,

# control = lmerControl(optimizer = "bobyqa",

# optCtrl = list(maxfun=2e6)))

#summary(res.2p.3way)$varcor

#mod_reduce2='rts.p ~ emotion * Label * language + (emotion * Label * language |participant) + (emotion + Label + language |item)'

#res.3p.3way=lmer(eval(parse(text=mod_reduce2)), data=dat_emotion_3way,

# control = lmerControl(optimizer = "bobyqa",

# optCtrl = list(maxfun=2e6)))

#summary(res.3p.3way)$varcor

#mod_reduce3='rts.p ~ emotion * Label * language + ((emotion + Label + language)^2 |participant) + (1|item)'

#res.4p.3way=lmer(eval(parse(text=mod_reduce3)), data=dat_emotion_3way,

# control = lmerControl(optimizer = "bobyqa",

# optCtrl = list(maxfun=2e6)))

#summary(res.4p.3way)$varcor

mod_reduce4='rts.p~ emotion * Label * language + (emotion + Label + language |participant) + (1|item)'

res.5p.3way=lmer(eval(parse(text=mod_reduce4)), data=dat_emotion_3way,

control = lmerControl(optimizer = "bobyqa",

optCtrl = list(maxfun=2e6)))

diag.vals <- getME(res.5p.3way,"theta")[getME(res.5p.3way,"lower") == 0]

any(diag.vals < 1e-6)

summary(res.5p.3way)

pprint(summary(res.5p.3way), "Main_lmerFit_NeutrialRemoval.html", type = "html")

###proficiency######

dat_emotion_English_proficiency <- subset(dat_emotion_3way, language=="English")

dat_emotion_English_proficiency$language <- droplevels(dat_emotion_English_proficiency$language)

str(dat_emotion_English_proficiency)

mod='rts.p ~ emotion * Label * proficiency_std + (1 |participant) + (1 |item)'

res.1p.3way=lmer(eval(parse(text=mod)), data=dat_emotion_English_proficiency,

control = lmerControl(optimizer = "bobyqa",

optCtrl = list(maxfun=2e6)))

summary(res.1p.3way)

pprint(summary(res.1p.3way), "Main_lmerFit_RTs_Proficiency.html", type = "html")

######Accuracy

dat_Summary_Accuracy <- ddply(dat_emotion, c("condition","language"), summarise,

N = length(Accuracy),

meanACC = mean(Accuracy),

sd = sd(Accuracy),

se = sd / sqrt(N)

)

dat_Summary_Accuracy

#keeping only for each language：Chinese

dat_emotion_Accuracy_Chinese <- subset(dat_emotion, language=="Chinese")

dat_emotion_Accuracy_Chinese$language <- droplevels(dat_emotion_Accuracy_Chinese$language)

str(dat_emotion_Accuracy_Chinese)

#repeated contrasts

contrasts(dat_emotion_Accuracy_Chinese$condition) <- contr.hypothesis(HcRep)

errors_test_Chinese <- lme4::glmer(Accuracy ~ condition +

(1|participant) + (1|item),

data = dat_emotion_Accuracy_Chinese,

family = "binomial"(link="logit"), control=glmerControl(optimizer="bobyqa", optCtrl=list(maxfun=1e6)))

summary(errors_test_Chinese)

pprint(summary(errors_test_Chinese), "Main_lmerFit_Chinese_Accuracy.html", type = "html")

#keeping only for each language：English

dat_emotion_Accuracy_English <- subset(dat_emotion, language=="English")

dat_emotion_Accuracy_English$language <- droplevels(dat_emotion_Accuracy_English$language)

str(dat_emotion_Accuracy_English)

#repeated contrasts

contrasts(dat_emotion_Accuracy_English$condition) <- contr.hypothesis(HcRep)

errors_test_English<- lme4::glmer(Accuracy ~ condition + proficiency_std +

(1|participant) + (1|item),

data = dat_emotion_Accuracy_English,

family = "binomial"(link="logit"), control=glmerControl(optimizer="bobyqa", optCtrl=list(maxfun=1e6)))

summary(errors_test_English)

pprint(summary(errors_test_English), "Main_lmerFit_English_Accuracy.html", type = "html")

# Second Step (remove neural)

dat_emotion_3way_accuracy <- subset(dat_emotion, condition !="neutral")

dat_emotion_3way_accuracy$condition <- droplevels(dat_emotion_3way_accuracy$condition)

dat_emotion_3way_accuracy$emotion <- fct_collapse(dat_emotion_3way_accuracy$condition, Negative = c("negativelb","negativeld"), Positive = c("positivelb","positiveld"))

dat_emotion_3way_accuracy$Label <- fct_collapse(dat_emotion_3way_accuracy$condition, Label = c("negativelb","positivelb"), Laden = c("negativeld","positiveld"))

str(dat_emotion_3way_accuracy)

dat_emotion_3way_accuracy_summary <- ddply(dat_emotion_3way_accuracy , c("participant", "emotion", "Label","language"), summarise,

N = length(Accuracy),

meanACC = mean(Accuracy),

sd = sd(Accuracy),

se = sd / sqrt(N)

)

dat_emotion_3way_accuracy_summary

sink("3_way_accuracy.txt")

print(dat_emotion_3way_accuracy_summary)

sink()

dat_emotion_3way_accuracy_summary %>%

ggplot(aes(x = Label, y = meanACC, color = emotion, group = interaction(participant, emotion))) +

geom_point(position = position_dodge(width = 0.2), size = 1, alpha = 0.18) +

geom_line(position = position_dodge(width = 0.2), size = 0.5, alpha = 0.18) +

stat_summary(aes(group = emotion), fun = mean, geom = "line", size = 1) +

stat_summary(aes(group = emotion), fun = mean, geom = "point", size = 2) +

scale_x_discrete(name = "") +

scale_y_continuous( "Accuracy [Logit]", breaks = seq(0.8,1, by = 0.1)) +

coord_cartesian(ylim = c(0.8, 1)) +

scale_color_manual(name = "", values = cbPalette) +

facet_grid( . ~ language, scales = "free")

ggsave(file = "Plot.png", width = 15, height = 6, units = "cm", dpi = 1000)

#sum contrast coding

dat_emotion_3way_accuracy$emotion_contra = ifelse(dat_emotion_3way_accuracy$emotion == "Positive", -0.5, 0.5)

dat_emotion_3way_accuracy$label_contra = ifelse(dat_emotion_3way_accuracy$Label == "Label", -0.5, 0.5)

dat_emotion_3way_accuracy$language_contra = ifelse(dat_emotion_3way_accuracy$language == "Chinese", -0.5, 0.5)

#Modelling

#errors_test_3way_full<- lme4::glmer(Accuracy ~ emotion_contra * label_contra * language_contra + (emotion_contra * label_contra * language_contra||participant) + (emotion_contra * label_contra * language_contra||item),

# data = dat_emotion_3way_accuracy,

# family = "binomial"(link="logit"), control=glmerControl(optimizer="bobyqa", optCtrl=list(maxfun=1e6)))

#summary(errors_test_3way)

errors_test_3way_reduced<- lme4::glmer(Accuracy ~ emotion_contra * label_contra * language_contra + (emotion_contra + label_contra + language_contra|participant) + (language_contra|item),

data = dat_emotion_3way_accuracy,

family = "binomial"(link="logit"), control=glmerControl(optimizer="bobyqa", optCtrl=list(maxfun=1e6)))

summary(errors_test_3way_reduced)

errors_test_3way<- lme4::glmer(Accuracy ~ emotion_contra * label_contra * language_contra + (emotion_contra + language_contra |participant) + (1|item),

data = dat_emotion_3way_accuracy,

family = "binomial"(link="logit"), control=glmerControl(optimizer="bobyqa", optCtrl=list(maxfun=1e6)))

summary(errors_test_3way)

errors_test_3way_Base<- lme4::glmer(Accuracy ~ emotion_contra * label_contra * language_contra + (1 |participant) + (1|item),

data = dat_emotion_3way_accuracy,

family = "binomial"(link="logit"), control=glmerControl(optimizer="bobyqa", optCtrl=list(maxfun=1e6)))

summary(errors_test_3way_Base)

anova(errors_test_3way_reduced, errors_test_3way, errors_test_3way_Base)

diag.vals <- getME(errors_test_3way_reduced,"theta")[getME(errors_test_3way_reduced,"lower") == 0]

any(diag.vals < 1e-6)

summary(errors_test_3way_reduced)

pprint(summary(errors_test_3way_reduced),"Main_lmerFit_NeutrialRemoval_accuracy.html", type = "html")

Anova_model <- car::Anova(errors_test_3way_reduced)

index=Anova_model$`Pr(>Chisq)` <= 0.1

# looking for p.val lower or equal to 0.1

effec=row.names(Anova_model[index,]); effec

sink('poshoc_accuracy.txt')

for(i in effec) {

cat('EFFECT (Accuracy):',i, fill=TRUE)

print(poshoc.fun(i, model=errors_test_3way_reduced))

}

sink()

# Once the significant or interesting effects of the anova summary table are

# determined, simple effect contrasts must be considered (for interactions).

# This involves (in the case of triples and quadruples) the choice of pivot

# factors on which to make the pair-wise comparisons. In order to facilitate

# this task I have created the post-hoc.fun function. This makes use of the

# emmeans package to first determine the marginal means and their standard

# errors and then make the comparisons. Since all factors have two levels,

# initially the contrast with or without correction against error type I give

# the same value of p

###############################################################################

poshoc.fun=function(which.effect, model){

which.effect=strsplit(which.effect, ':')[[1]]

emm_options(lmer.df = "asymptotic")

pred.mean=list(); poshoc=list()

if(length(which.effect)==1){

lab_=paste('~',which.effect)

pred.mean[[1]]=lsmeans::lsmeans(model,eval(parse(text=lab_)))

poshoc[[1]]=summary(pairs(pred.mean[[1]], adjust='hochberg'))

names(poshoc)[1]=lab_;

}

if(length(which.effect)==2){

for(i in 1:length(which.effect)) {

if(i==1) lab_=paste('~',which.effect[1],'|',which.effect[2]) else lab_=paste('~',which.effect[2],'|',which.effect[1])

pred.mean[[i]]=emmeans::emmeans(model, eval(parse(text=lab_)))

poshoc[[i]]=summary(pairs(pred.mean[[i]], adjust='hochberg'))

names(poshoc)[i]=lab_;

}

}

if(length(which.effect)==3){

for(i in 1:length(which.effect)) {

if(i==1) lab_= paste('~ ',which.effect[1],'|',which.effect[2],':',which.effect[3],sep='')

if(i==2) lab_= paste('~ ',which.effect[2],'|',which.effect[1],':',which.effect[3],sep='')

if(i==3) lab_= paste('~ ',which.effect[3],'|',which.effect[1],':',which.effect[2],sep='')

pred.mean[[i]]=emmeans::emmeans(model, eval(parse(text=lab_)))

poshoc[[i]]=summary(pairs(pred.mean[[i]], adjust='hochberg'))

names(poshoc)[i]=lab_;

}

}

return(poshoc)

}

#####PROFICINECY

dat_emotion_English_proficiency_ACC <- subset(dat_emotion_3way_accuracy, language=="English")

dat_emotion_English_proficiency_ACC$language <- droplevels(dat_emotion_English_proficiency_ACC$language)

str(dat_emotion_English_proficiency_ACC)

errors_test_3way_Base_proficiency<- lme4::glmer(Accuracy ~ emotion_contra * label_contra * proficiency_std + (1 |participant) + (1|item),

data = dat_emotion_English_proficiency_ACC,

family = "binomial"(link="logit"), control=glmerControl(optimizer="bobyqa", optCtrl=list(maxfun=1e6)))

summary(errors_test_3way_Base_proficiency)

pprint(summary(errors_test_3way_Base_proficiency), "Main_lmerFit_ACC_Proficiency.html", type = "html")

Anova_model2 <- car::Anova(errors_test_3way_Base_proficiency)

index=Anova_model2$`Pr(>Chisq)` <= 0.1

# looking for p.val lower or equal to 0.1

effec=row.names(Anova_model2[index,]); effec

sink('poshoc_accuracy_proficiency.txt')

for(i in effec) {

cat('EFFECT (Accuracy):',i, fill=TRUE)

print(poshoc.fun(i, model=errors_test_3way_Base_proficiency))

}

sink()

###############################################################################

##############################Power#############################################

library(MASS)

library(simr)

library(future)

library(future.apply)

Model <- errors_test_3way_Base

print(summary(Model),corr=F)

##define the basic parameters of the simulation: How many samples to run (1000) and what the α

#level for statistical significance is (0.05).

nsim <- 100

alpha <- 0.05

##identify which fixed effect we want to run the the simulation for and what effect size

##we are interested in (see the model summary above).

##This “effect size” refers to the unstandardized estimate for the effect (regression coefficient) as would be seen in the model summary.

teff <- "emotion_contra:label_contra:language_contra"

veff <- 0.07

##First we retrieve the fixed effects vector from the model and set the effect in question to the desired value.

fef <- fixef(Model)

fef[teff] <- veff

##Then we retrieve the random effects structure from the model and retain only the variance-covariance matrix,

##discarding the standard deviations and the correlation matrix.

vcv <- VarCorr(Model)

for (l in names(vcv)) {

attr(vcv[[l]],"stddev") <- NULL

attr(vcv[[l]],"correlation") <- NULL

}

##Finally recover the data frame from the model, retain only the independent variables and random factors from it

sdata <- cbind(Model@frame[,c("participant","item","emotion_contra","label_contra","language_contra")],

as.data.frame(model.matrix(Model)))

sdata <- Model@frame[,-1] # just exclude the dependent variable in the 1st column, the rest is already there

na.omit(sdata)

#Note that there should be no missing values in the data frame! Use na.omit() if passing a data frame with NA values in any of these variables.

#We also need to specify any optimizer parameters we want to be used with model fit.

glmerctrlist <- glmerControl(optCtrl=list(maxfun=1e5), optimizer = "bobyqa")

##We use all these components to build the model structure that will be used for the simulation.

##This also includes the model formula retrieved directly from the model object as well.

tglmer <- makeGlmer(attr(Model@frame,"formula"),

family="binomial", fixef=fef, VarCorr=vcv, data=sdata)

##Before runing the simulation, we set up R to use as many cores as we have available.

##On a regular windows PC we would just initialize a “multisession” plan as follows:

plan(multisession)

##On our HPC Linux cluster it turns out that we get best performance

##if we limit each PID to use one core only and run as many instances (PIDs) as the available cores:

Sys.setenv(OMP_NUM_THREADS=1)

plan(multisession,workers=64)

##The actual simulation is running using the following code.

##The trick that achieves parallel support for simr via future/future.apply was based on the discussion for simr issue #39 on github.

##Specifically, instead of running the desired number of simulations using function powerSim directly for Nsim samples, this trick runs the function repeatedly Nsim times,

##each time with a single sample.

##In this way each run can be allocated to a different instance on a different core, and the future framework then collects and assembles all output into a list.

pstests <- future_replicate(nsim, powerSim(tglmer, nsim=1, test=fixed(teff,"z"),

fitOpts=list(control=glmerctrlist),

progress = FALSE),

future.globals = c("powerSim","tglmer","teff","glmerctrlist"),

simplify = FALSE)

plan(sequential)

pvals <- sapply(pstests,function(x){x$pval})

print(round(sum(pvals<alpha)/length(pvals),2))
